# A study on information perception and engagement, emphasizing the essential role of E-clinics among Palestinian adolescents

**DOI:** 10.1371/journal.pone.0322220

**Published:** 2025-04-29

**Authors:** Nour Issa, Maysaa Nemer, Niveen Abu-Rmeileh

**Affiliations:** 1 Institute of Community and Public Health, Birzeit University, West Bank, Birzeit, Palestine; 2 Department of Public Health, College of Health Sciences, Qatar University, Doha, Qatar; American University of Beirut Medical Center, LEBANON

## Abstract

Adolescents increasingly rely on electronic platforms for health information, highlighting their rapid technological adoption among significant developmental changes. Utilizing E-clinics presents a promising approach to enhance their health outcomes. This study aimed to identify adolescents’ preferred health information sources, the challenges guiding their choices, and assess their willingness to use E-clinics. A cross-sectional survey was conducted among adolescents in grades 8–12 in governmental schools across the West Bank, Palestine. 646 questionnaires were distributed (69.5% females, 30.5% males; median age 16). Results highlighted family/friends (34.4%), HCPs (32.5%), and the Internet (23.7%) as primary sources of health information, with 91.2% of Internet users seeking health advice. Internet convenience (56.2%), abundant information (31.9%), and anonymity (7.6%) were valued by participants. Place of residence significantly (p < 0.001) influenced health information sources, with 94.4% in the center preferring the Internet. Three key factors emerged: trust in HCPs (p < 0.001) influenced reliance on them (76.7%), and willingness to use E-clinics for privacy (63.5%); distrust in healthcare services led to Internet reliance (32.7%) and E-clinic interest for privacy (30.4%); privacy concerns (p = 0.00) led unconcerned participants (52.9%) to rely on the Internet, and 51.6% believed E-clinics were private. Trust in HCPs and healthcare services influenced E-clinic anonymity perception, while location and privacy concerns affected E-clinic benefits perception. Findings suggest that E-clinics can improve accessibility to healthcare by filling the gaps left by traditional healthcare models while also addressing privacy issues. Incorporating them in healthcare systems that serve adolescents can improve trust, accessibility, and improve well-being.

## Introduction

Adolescence marks a transitional phase in an individual’s life, characterized by significant physical and cognitive transformations contributing to overall well-being and growth [1], where health-risk behaviors could potentially be initiated (such as smoking, inappropriate sexual behaviors, drug use, and physical inactivity) [2]. As a result of these developmental changes, adolescents often seek clarity about their health and bodies. Consequently, they actively seek answers and health-related information from various sources [3], including parents, healthcare providers (HCPs), friends, and the Internet [4]. Seeking health information is an activity that is undertaken to satisfy a query [5]. As a result, adolescents seek different sources to obtain health information regardless of its reliability, clarity, simplicity, and usefulness [6]. Various factors can elucidate why adolescents favor one information source over another, including trust, confidence in the information source, and accessibility to the information [7]. Data propose that the sources of health information favored by adolescents might not necessarily constitute their primary sources of such information [8]. The traditional sources of health information for adolescents, such as family and friends [9,10], books and educational materials [11], television and radio [12], and HCPs [9], are becoming inadequate and not satisfying, leading to an increased reliance on the Internet [13]. Studies indicate that adolescents primarily use the Internet to address their health-related needs [14]. Following this, seeking assistance from family ranks as the second source, and HCPs constitute the third source for obtaining health information [15]. The internet has become extensively utilized and a fundamental component of adolescents’ daily lives. Studies have indicated that internet usage is fast, convenient, and offers a treasure of information [16]. Additionally, it provides a secure and confidential source of health information [17,18]. Adolescents are turning to the Internet as their main source of health information due to concerns about confidentiality and the difficulties they face when seeking care through conventional methods [19]. In contrast, studies found that searching online health information represents unique obstacles, encompassing factors such as facing unfamiliar terminology, competing with filters meant to block content, and difficulty determining information quality [19]. Consequently, research indicates that higher consumption of online health information is not exclusive to younger age groups but also extends to individuals with enhanced health literacy levels [20].

In Palestine, adolescents make up 30% of the total population [21]. Existing literature indicates that adolescents in Palestine exhibit a lack of trust in HCPs, coupled with low satisfaction levels with the healthcare system. Consequently, they rely on alternative sources for health information such as the Internet, and engage in home-based treatment [22]. According to the Palestinian Central Bureau of Statistics (PCBS), 92.6% of Palestinian families have Internet service at home [23]. 92% of individuals above 10 years old can access the Internet from anywhere [23]. Based on data from The Arab Center for the Advancement of Social Media, 76% of Palestinian adolescents access the Internet [24]. While there have been advancements in primary healthcare services in Palestine, healthcare services specifically designed for adolescents in Palestine are still in the process of development, primarily due to factors like ongoing conflict and resource constraints [22]. Adolescents face a range of unique health challenges influenced by both social and political factors, particularly in regions with limited healthcare infrastructure and social constraints. In Palestine, traditional healthcare services often present challenges for adolescents, including concerns about confidentiality, social stigma, and limited accessibility to specialized adolescent healthcare services. Living under occupation and dealing with ongoing conflict significantly impacts their access to healthcare, education, and psychosocial support. A study found that frequent exposure to violence, trauma, and instability can lead to mental health issues such as anxiety, depression, and post-traumatic stress disorder (PTSD), yet mental health services remain under-resourced and stigmatized in the Palestinian society [25]. Movement limitations from checkpoints and blockades make accessing care difficult, especially from rural areas [26]. The healthcare system is strained, with damaged infrastructure [27], shortages in human resources, as many skilled healthcare professionals leave the area, and cultural taboos around sensitive health topics like reproductive health further discourage adolescents from seeking care, with limited resources hindering the development of specialized adolescent healthcare services [28]. Consequently, adolescents often turn to more accessible ways, such as the Internet, to seek health information and support during this critical developmental stage. Additionally, privacy concerns hold significant importance for adolescents in a conservative culture like Palestine. A study conducted by The United Nations Fund for Population Activities (UNFPA) on social norms and sexual health among Palestinian youth revealed that certain topics related to adolescent reproductive health can be uncomfortable to discuss due to cultural and religious reasons. Accordingly, adolescents may refrain from discussing these matters with their parents or HCPs, and thus search for health information through the Internet [29]. During their inquisitive adolescent years, young individuals might use the Internet with limited supervision, and the content they access, whether beneficial or detrimental in terms of health information, can potentially shape their behaviors and overall development. Research has found that appropriate internet use among adolescents can enhance their well-being. Conversely, the improper consumption of online health information may have adverse effects on their growth and maturation [30].

E-clinics, which provide virtual consultations and digital health services, have emerged as a promising solution to bridge the gap in adolescent healthcare access [31]. However, the adoption of E-clinics in Palestine presents unique infrastructural and systemic challenges. Limited Internet accessibility, disparities in digital literacy, and regulatory barriers pose significant obstacles to the successful implementation of these services [32]. Moreover, cultural perceptions of virtual healthcare and trust in digital platforms may influence adolescents’ willingness to engage with E-clinics. Understanding these factors is crucial for assessing the feasibility and effectiveness of E-clinics in improving adolescent healthcare access in Palestine.

As the Internet provides a vast array of health information sources, concerns have arisen regarding the quality of this information and the challenges adolescents may encounter in accessing reliable health-related content. However, there is a lack of comprehensive data regarding adolescents’ favored health information sources, how they share health information, and their inclination toward utilizing a confidential online source like E-clinic for health-related queries. Despite the limitations posed by the country’s available resources, directing interventions toward adolescents can prove to be cost-effective. Integrating various technologies and informational functions into E-clinic applications tailored for adolescents could bring benefits not only to the young population but also to researchers, the healthcare system, and web designers in Palestine.

Given these challenges, this study aims to explore the perception and engagement of Palestinian adolescents with E-clinics, identifying the factors that facilitate or hinder their adoption. By examining adolescents’ attitudes, usage patterns, and concerns regarding digital healthcare, the study seeks to provide valuable insights for policymakers, HCPs, and technology developers. The findings will contribute to the broader discussion on integrating digital health solutions into adolescent healthcare systems, particularly in resource-limited and culturally sensitive settings like the West Bank - Palestine.

We conducted a regression analysis to identify the preferred health information sources used by Palestinian adolescents, explore privacy concerns and their influence on source selection, examine adolescents’ trust in HCPs, and assess their openness to using online electronic resources, such as E-clinics, for accessing trustworthy health information. Our approach focuses on understanding key factors influencing adolescents’ health-seeking behavior. Given that the implementation of E-clinics is a novel concept in the West Bank-Palestine, our broader objective was to evaluate adolescents’ readiness to adopt such an initiative and explore potential solutions for effectively integrating E-clinics into the future development and improvement of adolescent healthcare services.

## Materials and methods

### Study design, approach, and participants’ selection

A cross-sectional survey was carried out targeting all adolescents who reside in the three regions of the West Bank – Palestine (North, Middle, and South), aged 15–19 years old, and who were enrolled as regular students at governmental schools according to the Palestinian Ministry of Education records for the academic year 2022/2023. The sample size was calculated using the Survey System version 11.0 Sample Size Calculator [33]. A sample population of 650 participants was obtained, including a 5% margin of error and applying a 95% confidence level. The participants were stratified based on various demographic characteristics such as age, gender, educational level, place of residence, and location. The list of the governmental schools in the West Bank - Palestine was acquired from the Palestinian Ministry of Education. Schools were subsequently chosen based on the grade levels, specifically selecting schools that encompassed students from the 8th to the 12th grades. To ensure a diverse study population, we included both boys’ and girls’ schools from the three regions. Importantly, all selected schools, along with their attending students, were included in the study, and a total of 646 questionnaires were distributed to the study sample.

### Research instrument and data collection

The study was conducted using an anonymous online survey to assess adolescents’ perceptions regarding obtaining health information from diverse sources. The structured questionnaire was made accessible via an online link and was disseminated to the participants through school electronic platforms on March 23, 2023, we concluded the data collection phase on July 23, 2023. The Comprehensive Health Department in the schools facilitated this distribution across various directorates in the West Bank, including three regions: North, Middle, and South. The survey reached students aged 15–19 years old, encompassing those from 8th to 12th grades. The survey tool/questionnaire was precisely constructed after a thorough review of the relevant questionnaires in the existing literature. Questions were subsequently adopted, adapted to the Palestinian context, and then translated into Arabic, the native language of the Palestinian population. We completed a pilot test of our questionnaire, refining it based on feedback to improve clarity, reliability, and relevance for the main study. The structured questionnaire consisted of seven main sections and 22 items. The first section started with an information sheet explaining the study idea and objectives, followed by consent to participation. The second section included the sociodemographic characteristics including age, gender place of residence (city, village, refugee camp), location, and educational background. The third section was designed to explore the relationship between adolescents and HCPs, specifically the level of trust in discussing health-related issues and its impact on seeking health information from various sources. The fourth section was organized to understand the diverse sources that adolescents utilize to search for health-related information. The fifth section probed the significance of privacy and confidentiality, which in turn influenced adolescents’ inclination to acquire health information from electronic sources. The last but one section was created to inquire about the characteristics of health information sources, including their accuracy and credibility. The final section evaluated adolescents’ inclination to use/rely on electronic clinics as confidential sources of health information. Some questions (in sections 3,5 and 7) provided five response choices: “strongly disagree,” “disagree,” “neutral,” “agree,” and “strongly agree,” assigned values of 1, 2, 3, 4, and 5, respectively, following the Likert five-point scale.

### Ethical considerations

This study was conducted in accordance with the ethical principles outlined in the Declaration of Helsinki [34]. All procedures involving human participants were approved by the relevant Ethics Review Committee at the Institute of Community and Public Health at Birzeit University, with reference number (2022 (5-1)), and informed consent was obtained from participants aged 15-19, in line with Birzeit University’s guidelines, which state that parental consent is not required for this age group, as this reflects the principle of respecting adolescents’ autonomy and decision-making capacity, recognizing their ability to understand the study and its risks and benefits. Participation was completely voluntary, and all collected data were treated with confidentially, ensuring that no names or specific identifying marks were registered. Only the research team had access to the collected data for analyzing it.

### Statistical analysis

Descriptive statistics were employed to analyze the qualitative variables (age, gender, place of residence, location, and educational background), presenting frequencies and percentages, while means and standard deviations were calculated for the quantitative variables (trust in HCPs and healthcare services, variables related to access to health information, internet usage, perceptions of the reliability of online health information, the ease of online information retrieval, and the utilization of alternative sources for health-related information). To find associations and correlations, bivariate analyses were carried out using the Chi-square test. We used the Chi-square test to assess the relationship between two categorical variables, as it is ideal for analyzing nominal or ordinal survey data and provides clear insights into observed versus expected frequencies. Additionally, a Stepwise multivariate regression was employed to select the most significant associations among a large set of variables. The regression model was employed to examine how independent variables influence a dependent variable. Given the large set of variables in our study, we aimed to control for confounders and measure the strength and direction of the relationships. The significant level was established at a p-value < 0.05, and the statistical analysis was performed using SPSS 28.0 (Statistical Package for Social Sciences) to analyze the data [35].

## Results

Our results revealed interesting perspectives of the adolescents in the West Bank - Palestine regarding their preference for health information sources shaped by digital advancements with a focus on utilizing E-clinic, as well as their understanding of the diverse dynamics influencing their decisions in accessing and interacting with health-related content.

### Demographic characteristics of the study sample

A total of 646 students, out of the 650 invited, participated in this study. The participants were predominantly females (69.5%), with boys making up the remaining 30.5%. Only four students declined to participate in the survey. The average age of participants was 15 years old, and the majority (60.5%) were above 16 years of age. Geographic distribution showed that 39.6% of participants lived in the southern governorates of the West Bank, while 35.6% and 24.8% lived in the northern and middle areas, respectively. The majority of participants (49.4%) lived in villages, while 8.6% resided in refugee camps. Table 1 illustrates a summary of descriptive statistics of our data set.

**Table 1:** General characteristics of the study sample (n = 646).

Variable		Number	%
1. **Demographic Characteristics**			
**Age**	≥ 16 years	391	60.5
	< 16 years	255	39.5
**Gender**	Female	449	69.5
	Male	197	30.5
**Residence**	South	256	39.6
	North	230	35.6
	Center	160	24.8
**Locality**	Village	319	49.4
	City	271	42
	Refugee camp	56	8.6
2. **Sources of Health-related information**			
**Preferred sources of Health-related information**	Family and friends	222	34.4
	Providers (Healthcare, social, and school)	210	32.5
	Internet	153	23.7
	Others	61	9.4
**Ever used the Internet for health-related tips**	Yes	589	91.2
	No	57	8.8
**Reasons for using the Internet as a source of health-related information**	Convenience	363	56.2
	Availability of information	206	31.9
	Privacy	49	7.6
	Others	28	4.3
3. **Adolescents’ trust and privacy concerns**			
**Trust in HCPs**	Agree	406	62.8
	Disagree	132	20.5
	Not sure	108	16.7
**Trust in healthcare services**	Agree	381	59.0
	Disagree	150	23.2
	Not sure	115	17.8
**Privacy concerns**	Disagree	307	47.5
	Agree	232	35.9
	Not sure	107	16.6
4. **Perceptions on utilizing E-clinics**			
**E-clinics ensure privacy**	Agree	345	53.4
	Disagree	178	27.6
	Not sure	123	19.0
**E-clinic is beneficial**	Agree	600	92.9
	Disagree	46	7.1
	Not sure	0	0
**Using E-clinic takes time and effort**	Agree	307	47.5
	Disagree	232	35.9
	Not sure	107	16.6

### Sources of health-related information

#### Preferred information sources among adolescents: Media and non-media options.

Family and friends emerged as the top choice, with 34.4% of participants selecting it as their major source. HCPs, social workers, and school health counselors were the second most preferred sources, with 32.5% of participants indicating their trust in them for health information. Interestingly, the Internet (23.7%) was nearly as popular as HCPs. Surprisingly, 9.4% of participants sought information from sources other than the Internet, family, friends, HCPs, and school counselors, such as books, magazines, TV, and radio.

#### Reasons for utilizing the Internet as a health information source.

A large majority (91.2%) of participants reported using the Internet for health-related advice, with 56.2% finding it most convenient. Additionally, 31.9% appreciated the abundance of information available, while 7.6% noted that the anonymity provided by online platforms encouraged them to seek health information online. A small fraction (4.3%) used the Internet for purposes other than health information retrieval (Table 1).

### Adolescents’ trust and privacy concerns

#### Trust in HCPs and health services among adolescents.

Over half of the participants (62.8%) trusted HCPs and 59.0% trusted healthcare services. Only 20.4% and 23.2% never trusted HCPs and healthcare services, respectively.

#### Privacy concerns among adolescents.

35.9% of participants expressed concerns about sharing their personal and medical information. However, 47.5% of participants were relatively unconcerned about their privacy. A moderate portion (16.6%) remained uncertain about their privacy concerns (Table 1).

### Adolescents’ perception of utilizing E-clinic

The analysis of the data unveiled that 53.4% of participants expressed confidence in the E-clinic, believing that it ensures privacy for personal information and health queries. Conversely, only 27.6% of participants hold a contrary view. A significant proportion (92.9%) finds the use of E-clinic highly beneficial, while a small fraction (7.1%) holds a different opinion. Interestingly, 47.5% of participants feel that utilizing such a platform may be time-consuming and require substantial effort, whereas 35.9% believe the opposite (Table 1).

### The relationship between demographic characteristics, trust in HCPs, and privacy concerns among adolescents

#### The impact of demographic characteristics on trust in HCPs and healthcare services.

The analysis revealed that the association between demographic characteristics and trust in HCPs was slightly different but not to a statistically significant level (Table 2).

**Gender and age:** Gender exhibited a slight difference (p = 0.156), with girls showing a higher level of trust in HCPs than their counterparts, and an increase in predicted trust in HCPs for each additional year of age was observed (p = 0.204).

#### Geographical location and trust in HCPs and healthcare services.

In terms of locality (p = 0.198), 63.1% of participants residing in cities showed higher trust in HCPs compared to those in other localities. Furthermore, individuals residing in the southern areas (65.6%) demonstrated a higher likelihood of trusting HCPs (p = 0.149). The results showed that there is no significant association between adolescents’ trust in healthcare services and any of the demographic variables, except residence (p = .009), 63.7% of the participants who resided in the southern West Bank exhibited a higher level of trust in healthcare services than others.

#### The impact of demographic characteristics on adolescents’ privacy concerns.

The Chi-square test revealed no statistically significant association between adolescent privacy concerns and demographic factors.

**Gender and age:** It was observed that 37.1% of boys demonstrated heightened privacy concerns (p = 0.211). Among participants aged 16 and above, 37.1% exhibited an increased concern for privacy, while 48.1% displayed decreased concern.

#### Geographical location and privacy concerns.

The locality and residence factors were not significant (p = 0.175). 37.9% of those residing in villages had higher privacy concerns, and 40.6% living in the southern region exhibited an elevated level of privacy concern.

**Table 2:** The relationship between demographic characteristics, trust in HCPs and privacy concerns among adolescents (n = 646).

Variables		Gender				Age				Locality					Residence				
		Female	Male	Test statistic*	P	< 16	≥ 16	Test statistic*	P	City	Village	Refugee camp	Test statistic*	P	North	Center	South	Test statistic*	P
**1. Trust in HCPs**	**Disagree** **N (%)**	84 (18.7)	48 (24.4)	3.721	.156	45 (17.6)	87 (22.3)	3.178	.204	56 (20.7)	59 (18.5)	17 (30.4)	6.010	.198	52 (22.6)	40 (25.0)	40 (15.6)	6.756	.149
	**Not sure** **N (%)**	81 (18.0)	27 (13.7)			49 (19.2)	59 (15.1)			44 (16.2)	59 (18.5)	5 (8.9)			35 (15.2)	25 (15.6)	48 (18.8)		
	**Agree** **N (%)**	284 (63.3)	122 (61.9)			161 (63.1)	245 (62.7)			171 (63.1)	201 (63.0)	34 (60.7)			143 (62.2)	95 (59.4)	168 (65.6)		
**2. Trust in healthcare services**	**Disagree** **N (%)**	95 (21.2)	55 (27.9)	3.566	.169	50 (19.6)	100 (25.6)	3.653	.161	62 (22.9)	71 (22.3)	17 (30.4)	1.915	.751	56 (24.3)	51 (31.9)	43 (16.8)	13.517	.009*
	**Not sure** **N (%)**	83 (18.5)	32 (16.2)			51 (20.0)	64 (16.4)			48 (17.7)	57 (17.9)	10 (17.9)			37 (16.1)	28 (17.5)	50 (19.5)		
	**Agree** **N (%)**	271 (60.4)	110 (55.8)			154 (60.4)	227 (58.1)			161 (59.4)	191 (59.9)	29 (51.8)			137 (59.6)	81 (50.6)	163 (63.7)		
**3. Privacy Concerns**	**Disagree** **N (%)**	208 (46.3)	99 (50.3)	3.115	.211	119 (46.7)	188 (48.1)	2.233	.328	138 (50.9)	141 (44.2)	28 (50.0)	6.339	.175	114 (49.6)	81 (50.6)	112 (43.8)	6.339	.175
	**Not sure** **N (%)**	82 (18.3)	25 (12.7)			49 (19.2)	58 (14.8)			41 (15.1)	57 (17.9)	9 (16.1)			35 (15.2)	32 (20.0)	40 (15.6)		
	**Agree** **N (%)**	159 (35.4)	73 (37.1)			87 (34.1)	145 (37.1)			92 (33.9)	121 (37.9)	19 (33.9)			81 (35.2)	47 (29.4)	104 (40.6)		

* **Chi-square test.**

Data shown in numbers represent the number of participants. The number between brackets indicates the percentages equivalent.

*p < 0.05; **p < 0.01; ***p < 0.001.

### The relationship between health-related information sources, Internet use, and utilizing E-clinics, and the demographic characteristics among adolescents

The study examined associations between demographic characteristics and health-related information sources, as well as perceptions of using E-clinic among adolescents (Table 3).

**Table 3:** The relationship between sources, internet use, and utilizing E-clinics, and the demographic characteristics among adolescents (n = 646).

Variables		Gender				Age				Locality					Residence				
		Female	Male	Test statistic*	P	< 16	≥16	Test statistic*	P	City	Village	Refugee camp	Test statistic*	P	North	Center	South	Test statistic*	P
**Preferred sources of Health-related information**	**Family and friends** **N (%)**	145 (32.3)	77 (39.1)	3.52	.319	94 (36.9)	128 (32.7)	2.272	.581	103 (38.0)	93 (29.2)	26 (46.4)	11.685	.069	82 (35.7)	49 (30.6)	91 (35.5)	7.538	.274
	**Providers (Healthcare, social, and school)** **N (%)**	153 (34.1)	57 (28.9)			75 (29.4)	135 (34.5)			75 (27.7)	119 (37.3)	16 (26.6)			81 (35.2)	51 (31.9)	78 (30.5)		
	**Internet** **N (%)**	110 (24.5)	43 (21.8)			63 (24.7)	90 (23.0)			67 (24.7)	76 (23.8)	10 (17.9)			52 (22.6)	45 (28.1)	56 (21.9)		
	**Others** **N (%)**	41 (9.1)	20 (10.2)			23 (9.0)	38 (9.7)			26 (9.6)	31 (9.7)	4 (7.1)			15 (6.5)	15 (9.4)	31 (12.1)		
**Ever used the Internet for health-related tips**	**Yes** **N (%)**	412 (91.8)	177 (89.8)	0.622	.430	230 (90.2)	359 (91.8)	0.503	.478	253 (93.4)	283 (88.7)	53 (94.6)	4.842	.89	197 (85.7)	151 (94.4)	241 (94.1)	13.555	< 0.001**
	**No** **N (%)**	37 (8.2)	20 (10.2)			25 (9.8)	32 (8.2)			18 (6.6)	36 (11.3)	3 (5.4)			33 (14.3)	9 (5.6)	15 (5.9)		
**E-clinics ensure privacy**	**Disagree** **N (%)**	125 (27.8)	53 (26.9)	6.18	.045*	67 (26.3)	111 (28.4)	0.362	.835	73 (26.9)	89 (27.9)	16 (28.6)	6.031	.197	72 (31.3)	25 (15.6)	81 (31.6)	22.768	< 0.001**
	**Not sure** **N (%)**	96 (21.4)	27 (13.7)			50 (19.6)	73 (18.7)			43 (15.9)	72 (22.6)	8 (14.3)			38 (16.5)	27 (16.9)	58 (22.7)		
	**Agree** **N (%)**	228 (50.8)	117 (59.4)			138 (54.1)	207 (52.9)			155 (57.2)	158 (49.5)	32 (57.1)			120 (52.2)	108 (67.5)	117 (45.7)		
**E-clinic is beneficial**	**Disagree** **N (%)**	34 (7.6)	12 (6.1)	0.4054	.5	19 (7.5)	27 (6.9)	0.69	.792	16 (5.9)	25 (7.8)	5 (8.9)	1.131	.568	20 (8.7)	4 (2.5)	22 (8.6)	6.868	.032*
	**Agree** **N (%)**	415 (92.4)	185 (93.9)			236 (92.5)	364 (93.1)			255 (94.1)	294 (92.2)	51 (91.1)			210 (91.3)	156 (97.5)	234 (91.4)		
**Using E-clinic takes time and effort**	**Disagree** **N (%)**	159 (35.4)	73 (37.1)	3.115	.211	87 (34.1)	145 (37.1)	2.233	.328	92 (33.9)	121 (37.9)	19 (33.9)	2.859	.582	81 (35.2)	47 (29.4)	104 (40.6)	6.339	.175
	**Not sure** **N (%)**	82 (18.3)	25 (12.7)			49 (19.2)	58 (14.8)			41 (15.1)	57 (17.9)	9 (16.1)			35 (15.2)	32 (20.0)	40 (15.6)		
	**Agree** **N (%)**	208 (46.3)	99 (50.3)			119 (46.7)	188 (48.1)			138 (50.9)	141 (44.2)	28 (50.0)			114 (49.6)	81 (50.6)	112 (43.8)		

* **Chi-square test.**

Data shown in numbers represent the number of participants. The number between brackets indicates the percentages equivalent.

*p < 0.05; **p < 0.01; ***p < 0.001.

#### Geographical location and nternet usage.

A significant association (p < 0.001) was observed between place of residence and Internet usage. Participants residing in the center (94.4%) demonstrated a greater interest in utilizing the Internet for health-related information.

#### Demographic characteristics and utilizing E-clinics.

Regarding E-clinics, a significant association was found (p = 0.045). Boys (59.4%) were more inclined towards E-clinics due to privacy features.

#### Geographical location and utilizing E-clinics.

A significant association based on place of residence (P < 0.001) indicates that participants from the center (67.5%) perceived E-clinics as ensuring privacy more than those in other regions. Another significant association (p = 0.032) was identified, showing that participants residing in the center (97.5%) were most likely to agree with the benefits of E-clinics.

#### Geographical location and health information sources.

While no significant associations were found (p = 0.069) between locality and health information sources, it was shown that participants from refugee camps (46.4%) relied more on family and friends as a primary source and the HCPs (26.6%) as the second source. Additionally, though residence didn’t significantly impact health information sources (p = 0.274), northern region residents relied more on family (35.7%) than the Internet (22.6%).

#### Demographic characteristics and health-related information sources.

Boys showed a stronger trust in family and friends (39.1%) and the HCPs (28.9%) compared to girls (p-value for gender = 0.319). Age didn’t significantly influence information sources (p = 0.581), but older participants (34.5%) prefer HCPs, and younger participants (36.9%) rely on families and friends.

#### The impact of demographic characteristics and geographical location on understanding the practical use of E-clinics.

Concerning the perception that E-clinics are time-consuming, no significant associations were found, but trends existed such for the place of residence (p = 0.175). Participants from the South disagreed more (40.6%) compared to those from the North (35.2%). The P-value for gender was also not significant (p = 0.211), but 37.1% of boys believe that E-clinic is effortless and time-saving compared to 35.4% of girls. Locality didn’t significantly influence this perception, but residents in villages disagreed more (37.9%) compared to their counterparts (33.9%). Similarly, no significant association was found between age and E-clinic usage (p = 0.328), 37.1% of participants above age 16 believe E-clinic is easy to use compared to 48.1% within the same age group who agree it requires effort (Table 3).

### The associations between privacy and trust concerns, preferred sources of health information, using the Internet, and issues concerned with utilizing E-clinic

#### The relationship between trust in healthcare providers, health information sources, and E-clinic utilization.

Significant associations (p < 0.001) emerged between trust in HCPs and preferred health information sources. 76.7% of participants trusting HCPs were more likely to rely on them for health information, 60.8% turned to their families, and 49.0% relied on the Internet. 63.5% of those who trust HCPs also believe that utilizing E-clinics ensures privacy (p < 0.001).

#### The relationship between trust in healthcare services, health information sources, and E-clinic utilization.

A significant association (p < 0.001) also existed between trust in healthcare services and preferred health information sources. Among those lacking trust in healthcare services 32.7% favor the Internet, and those trusting healthcare services rely more on HCPs (70.0%). Similarly, beliefs about E-clinics’ privacy assurance were significantly (p < 0.001) linked to trust in healthcare services. 55.1% of participants who trust healthcare services also believe that E-clinics ensure privacy, while 30.4% of those who don’t trust healthcare services still believe that E-clinics ensure privacy, as shown in Table 4.

**Table 4:** The associations between privacy and trust concerns, and preferred sources of health information (n = 646).

Variables			Preferred source of health information			Test statistic*	P-Value	Ever used the Internet for health-related tips		Test statistic*	P-Value		E-clinics ensure privacy		Test statistic*	P-Value		E-clinic is beneficial		Test statistic*	P-Value
		Internet	Family	Providers	Other sources			Yes	No			Disagree	Not sure	Agree			Disagree	Not sure	Agree		
**Trust in HCPs**	**Disagree** **N (%)**	48 (31.4)	50 (22.5)	19 (9.0)	15 (24.6)	36.32	< .001***	123 (20.9)	9 (15.8)	1.001	.606	35 (19.7)	13 (10.6)	84 (24.3)	26.22	< .001***	12 (26.1)	0	120 (20.0)	3.609	.165
	**Not sure** **N (%)**	30 (19.6)	37 (16.7)	30 (14.3)	11 (18.0)			99 (16.8)	9 (15.8)			29 (16.3)	37 (30.1)	42 (12.2)			11 (23.9)	0	97 (16.2)		
	**Agree** **N (%)**	75 (49.0)	135 (60.8)	161 (76.7)	35 (57.4)			367 (62.3)	39 (68.4)			114 (64.0)	73 (59.3)	219 (63.5)			23 (50.0)	0	383 (63.8)		
**Trust in healthcare services**	**Disagree** **N (%)**	50 (32.7)	59 (26.6)	27 (12.9)	14 (23.0)	23.873	< .001***	142 (24.1)	8 (14.0)	3.742	.154	29 (16.3)	16 (13.0)	105 (30.4)	42.892	< .001***	13 (28.3)	0	137 (22.8)	1.65	.438
	**Not sure** **N (%)**	26 (17.0)	41 (18.5)	36 (17.1)	12 (19.7)			106 (18.0)	9 (15.8)			24 (13.5)	41 (33.3)	50 (14.5)			10 (21.7)	0	105 (17.5)		
	**Agree** **N (%)**	77 (50.3)	122 (55.0)	147 (70.0)	35 (57.4)			341 (57.9)	40 (70.2)			125 (70.2)	66 (53.7)	190 (55.1)			23 (50.0)	0	358 (59.7)		
**Privacy Concerns**	**Disagree** **N (%)**	81 (52.9)	111 (50.0)	83 (39.5)	32 (52.5)	14.145	.028*	289 (49.1)	18 (31.6)	8.232	.016*	86 (48.3)	43 (35.0)	178 (51.6)	38.107a	0***	12 (26.1)	0	295 (49.2)	12.001	0.002**
	**Not sure** **N (%)**	23 (15.0)	33 (14.9)	36 (17.1)	15 (24.6)			98 (16.6)	9 (15.8)			24 (13.5)	43 (35.0)	40 (11.6)			7 (15.2)	0	100 (16.7)		
	**Agree** **N (%)**	49 (32.0)	78 (35.1)	91 (43.3)	14 (23.0)			202 (34.3)	30 (52.6)			68 (38.2)	37 (30.1)	127 (36.8)			27 (58.7)	0	205 (34.2)		

*Chi square test.

Data shown in numbers represent the number of participants. The number between brackets indicates the percentages equivalent.

*p < 0.05; **p < 0.01; ***p < 0.001.

#### The relationship between privacy, health information sources, using the Internet and utilizing E-clinics.

Privacy concerns showed significant associations with health information sources p = 0.028, Internet usage p = 0.016, E-clinics ensure privacy p = 0.000 and E-clinics are beneficial p = 0.002. Those who don’t prioritize privacy tend to trust the Internet more (52.9%) and use HCPs less (39.5%) for health-related information compared to 43.4% who were concerned about privacy and also consider HCPs their primary source of health information. For those unconcerned about privacy, 49.1% use the Internet for health-related tips, while 34.3% of those who prioritize privacy also use the Internet for health-related advice (Table 4). A significant association (p = 0.00) between privacy concerns and E-clinics as a private platform was evident. Even though 51.6% of participants were unconcerned about privacy, they still believe that E-clinics are secure. Conversely, 36.8% of those who prioritize privacy believed in E-clinics’ security (Table 4). Lastly, a significant association (p = 0.002) was found between privacy concerns and the perceived benefits of E-clinics. Notably, 58.7% of those prioritizing privacy believe that using E-clinics is not beneficial, while 49.2% of those unconcerned about privacy still believe in the benefits of E-clinics (Table 4).

#### Trust in healthcare providers and services as predictors of perceived privacy in E-clinics.

The multivariate analysis revealed that trust in both HCPs and healthcare services emerged as the most influential predictors of adolescents’ perception of E-clinics’ privacy assurance, better than privacy concerns. Participants who strongly expressed distrust in HCPs were more likely to disagree that E-clinics ensure privacy (p = 0.012). Similarly, those lacking trust in healthcare services were also more likely to strongly disagree that E-clinics ensure privacy (p < 0.001), as shown in Table 5.

**Table 5:** Multivariate logistic regression model predictors of E-clinics ensure privacy and E-clinics is beneficial.

E-clinics ensure privacy			N	Odds ratio	95% Confidence interval	P-value
**Disagree**	**Privacy concern**	**Disagree**	29	0.97	(0.649, 1.449)	0.882
		**Not sure**	24	1.155	(0.637, 2.095)	0.636
		**Agree**	125	(REF)	(REF)	(REF)
	**Trust in HCPs**	**Disagree**	29	2.551	(1.233, 5.275)	0.012
		**Not sure**	24	1.878	(1.018, 3.464)	0.044
		**Agree**	125	(REF)	(REF)	(REF)
	**Trust in healthcare services**	**Disagree**	86	0.214	(0.102, 0.448)	< 0.001
		**Not sure**	24	0.503	(0.269, 0.941)	0.031
		**Agree**	68	(REF)	(REF)	(REF)
**Not sure**	**Privacy concern**	**Disagree**	13	0.908	(0.548, 1.505)	0.707
		**Not sure**	37	3.24	(1.808, 5.806)	0
		**Agree**	73	(REF)	(REF)	(REF)
	**Trust in healthcare provider**	**Disagree**	16	0.807	(0.324, 2.011)	0.646
		**Not sure**	41	1.94	(1.032, 3.648)	0.04
		**Agree**	66	(REF)	(REF)	(REF)
	**Trust in healthcare services**	**Disagree**	43	0.538	(0.23, 1.254	0.151
		**Not sure**	43	1.444	(0.781, 2.67)	0.241
		**Agree**	37	(REF)	(REF)	(REF)

#### Influence of geographic location and privacy concerns on perception of E-clinic benefits.

The participant’s place of residence and their privacy concerns were found to be the strongest predictors influencing their perception of the advantages of E-clinics. Residents of the southern and northern regions strongly believe that E-clinics offer benefits, with those in the south demonstrating a stronger influence. Similarly, individuals who had privacy concerns and were uncertain about them were stronger predictors of believing that E-clinics are beneficial, with those who were uncertain showing a greater impact than those who agreed (Table 6).

**Table 6:** Logistic regression model predictors of E-clinic is beneficial.

Place of residence	Covariate	N	Odds ratio	95% Confidence interval	P-value
**Southern governorates**	256	3.339	(1.122, 9.942)	0.03
**Central governorates**	160	0.927	(0.488, 1.759)	0.816
	**Northern governorates**	230	(Ref)	(Ref)	(Ref)
**Privacy concerns**	**Disagree**	307	1.738	(0.727, 4.155)	0.214
	**Not sure**	107	3.113	(1.534, 6.314)	0.002
	**Agree**	232	(Ref)	(Ref)	(Ref)

*Agree is the reference.

## Discussion

Given the widespread use of technology and digitalization in adolescents’ lives, it was common for the participants to rely on the Internet for health-related issues. The study examined the factors that might affect adolescents’ choice of health information, including their age, gender, place of residence, and locality. Additionally, the study delved into specific issues such as trust in healthcare professionals and privacy concerns to gain a deeper understanding of their inclination to use E-clinics.

### Trust and geographical location as key predictors of adolescents’ health information preferences

Trust and geographical location were generally found as key predictors in shaping adolescents’ preference for health information sources and their perception regarding utilizing E-clinics, outweighing privacy concerns. The participants reported being unconcerned about their privacy. This may be attributed to a lack of awareness regarding their rights to protect personal and health information, as observed in other studies [36,37]. Adolescents’ trust in HCPs is shaped by confidentiality, honesty, respect, and empathy, which encourage help-seeking behaviors [38]. This trend is observed globally, with studies from the U.S. and UK emphasizing the role of interpersonal trust in accessing care [39,40]. Moreover, healthcare access varies by region, with rural areas, especially in Africa and Asia, facing infrastructure challenges, while urban areas offer better digital access but heightened privacy concerns. Cultural differences also play a role, with some societies prioritizing family involvement, like in some parts of Africa, and others emphasizing individual autonomy, like in Western countries [41]. Privacy awareness differs globally, affecting adolescents’ concerns about data protection. While developed regions with strong legal frameworks like Europe report higher privacy concerns, less developed areas often show lower awareness [42], which may explain reduced concern, as seen in our study.

### Variability of trust in HCPs across regions and its influencing factors

Adolescents generally exhibited a high level of trust in HCPs and services despite their age, sex, and locality, emphasizing the variability of this relationship influenced by factors like honesty, confidentiality, and communication [43], contrary to findings in the Gaza Strip where distrust stemmed from concerns regarding privacy and inadequate service delivery tailored for adolescents [22]. The participants who trusted HCPs relied on them as their primary health information source, emphasizing reliance on professional guidance. Despite this, a substantial portion of participants also turned to their families and friends, highlighting family influence and interpersonal relationships in searching for health information [4,44]. This aligns with another study suggesting that both girls and boys communicate more openly with their families and rely on them for health information [45]. However, when traditional sources such as family and HCPs fall short, adolescents turn to online sources for health information despite privacy concerns, indicating the evolving role of online resources because they are convenient, ensure privacy and anonymity, and offer a wealth of information [46–48]. This behavior stems from the belief that the benefits outweigh the risks, supported by easy access through smartphones and computers [4]. Although gender didn’t significantly affect adolescents’ preference for health information sources, boys in our study exhibited proficiency in searching online for health information, consistent with previous studies suggesting that girls have potential Internet literacy issues and lack of reaching skills [49,50]. Interestingly, the participation rate of girls in our study was higher than that of boys. This could be due to their greater involvement in academic health discussions and a stronger interest in health-related topics, influenced by societal expectations that encourage higher participation [51]. Additionally, girls may have been more present or encouraged to participate in the schools where the survey was conducted. In the Palestinian context, particularly in villages with high response rates, cultural norms often keep girls at home more than boys, who tend to engage in outdoor activities [52]. This cultural dynamic likely contributed to the higher responsiveness among girls in our study.

### Geographical location and its influence on trust in healthcare services and Internet reliance

Adolescents’ trust in healthcare services is influenced by geographical location. Rural areas often face barriers such as limited access, cultural stigma, and confidentiality concerns, leading to lower trust levels [53]. In East Africa, rural adolescents struggle with distance from health facilities and cultural stigma, whereas urban areas with better infrastructure tend to report higher trust [54]. In our study the geographical location held equal importance to trust; adolescents residing in the south trust healthcare services more frequently. This trust could be attributed to the unique nature of the area and the availability of healthcare services as well as the relationship between HCPs and adolescents. Contrary to findings in previous studies, which suggested that adolescents in rural areas typically exhibit lower levels of trust due to factors such as rural culture, issues with confidentiality and anonymity, challenges in accessing healthcare services, and negative past experiences [55–57]. Adolescents who distrust traditional healthcare services often seek health information online, a global trend where Internet access is available. The study participants who distrust healthcare services tend to rely on the Internet. Prior research aligns, suggesting that distrustful adolescents seek online alternatives, especially in culturally sensitive topics, despite potential reliability concerns [58,59]. In developed countries like the U.S., strong digital literacy influences reliance on online health resources, while in less developed regions like Africa, limited digital infrastructure restricts access but also presents opportunities for mobile health solutions [53].

### Trust, digital healthcare, and adolescent preferences for health information access

The significant association between trust in HCPs and confidence in E-clinics’ privacy underscores the complicated interplay between trust and confidentiality perceptions in digital healthcare platforms. Adolescents in some regions may exhibit skepticism towards digital platforms, fearing inadequate privacy protections compared to traditional services. A study conducted in the United States found that adolescents who had strong relationships with their HCPs were more likely to utilize digital health resources, as they felt assured of their privacy and the quality of care received [60]. Participants who trust HCPs also believe in E-clinics’ privacy assurances, indicating a fusion of traditional healthcare trust with confidence in technological protection. They eagerly utilize digital platforms to communicate with HCPs, strengthening the patient-provider relationship and accessing reliable information [61]. While many adolescents embrace digital health tools, they often prefer in-person visits for sensitive issues. Studies show adolescents’ willingness to use technology for health improvement but also note that some prefer in-person visits for face-to-face interaction and confidentiality concerns [62,63]. A Canadian study found that despite valuing digital convenience, adolescents favored personal interactions for confidential matters [61]. Similarly, the participants in our study who lacked trust in HCPs and healthcare services were less likely to believe in E-clinics’ privacy. A study found that low trust in HCPs can hinder adolescents’ confidence in E-clinics, thus losing trust to seek health information [64]. In Europe, a study highlighted that adolescents often prefer face-to-face interactions due to concerns about confidentiality and the perceived impersonal nature of online consultations [60]. This reflects a broader trend where cultural attitudes towards technology and healthcare influence trust dynamics.

### Adolescents’ privacy perceptions and role of geographical location in shaping trust in E-clinics and its benefits

Adolescents, despite distrust in healthcare services, may perceive E-clinics as offering privacy, possibly due to negative experiences or perceptions of HCPs [37,65]. Confidence in digital health technologies’ privacy measures may bolster trust in the E-clinics’ security [66]. Those who were uncertain about their privacy concerns exhibited a greater impact on their perception of E-clinics’ benefits compared to those who simply agreed with privacy concerns. A study found that adolescents may have concerns about the privacy of their health information while using E-clinics especially if they believe that their privacy might be compromised [64,67].

Furthermore, geographical location also played a role, with southern region adolescent residents exhibiting a stronger influence on the perception of the benefits of E-clinic. A study found that geographical disparities can influence adolescents’ beliefs about the benefits offered by E-clinics [67]. Those residing in the center viewed digital platforms as ensuring private conversations with HCPs. This reflects cultural taboos around sensitive topics, where discussing such matters openly remains challenging and highlights adolescents’ perception of E-clinics as safe spaces for open conversations [68]. Therefore, there was no doubt that the adolescents in the center perceived the benefits of E-clinics likely influenced by their heavy reliance on digital platforms for health information.

While the findings highlight the potential benefits of E-clinics, successful adoption among Palestinian adolescents requires addressing key barriers, particularly trust and privacy concerns. Clear communication about data protection policies, including how personal information is stored and used, can help alleviate fears of misuse. Providing adolescents with control over their health data, such as allowing them to access and manage their records securely, can further enhance trust. Additionally, integrating digital literacy and privacy education into school curricula can empower young users to navigate online healthcare safely. Engaging parents and community leaders in awareness campaigns can also foster a supportive environment, reinforcing the legitimacy of E-clinics as a reliable healthcare option. By integrating these measures, E-clinics can become a reliable and accepted healthcare solution for adolescents in Palestine.

### Summary

Our results were consistent with previous studies that have shown that the digital transformation of healthcare has disrupted the relationship between adolescents and HCPs, leading to the emergence of E-clinics and other digital healthcare technologies that offer new opportunities for healthcare information [69,70]. This transition reflects the evolving landscape of healthcare, where digital platforms offer new avenues for health information and improve health outcomes. Despite a notable portion lacking trust in HCPs, many still rely on E-clinics for privacy, suggesting a shift influenced by various factors like past experiences and attitudes toward technology.

Our findings emphasize the complexity of adolescents’ perception of utilizing E-clinics to seek health information, influenced by personal trust in HCPs, family factors, the changing environment of digital health resources, and the emergence of electronic healthcare platforms. Recognizing these findings is important for customizing interventions and communication approaches designed to encourage adolescents to seek health information from reliable sources and improve their overall well-being.

Despite valuable insights from this study, data collection faced limitations due to extended strikes in governmental schools. Surveys had to be distributed electronically, awaiting the strike’s end. Another limitation is the higher proportion of females compared to males in the study population, potentially affecting the generalizability of the results. The study also focused on governmental schools for a uniform sample excluding private schools which may cause selection bias, as their students often have greater access to digital health resources, affecting the generalizability of our findings. Additionally, building on our previous qualitative research Adolescent–Provider Relationship and Perception of Protection of Health Data: Insights from the West Bank – Palestine, DOI: (https://doi.org/10.1080/02673843.2024.2420673), published on November 3, 2024, may emphasize adolescents already inclined toward digital health solutions. A key strength lies in the comprehensive quantitative study across diverse regions in the West Bank, ensuring broad applicability. Future studies should include diverse educational settings and varied adolescent groups to enhance applicability.

### Recommendations

The practical implementation of E-clinics in Palestine must address both the technological infrastructure and the unique healthcare needs of the population, particularly adolescents. This includes ensuring a reliable Internet infrastructure and developing user-friendly digital platforms for consultations. Additionally, HCPs should be trained in virtual care, and a diverse range of health services should be offered, including primary healthcare, health education, and mental health support. It is essential to establish strong data protection measures and to educate users about privacy. Services should be integrated with schools, and mobile health units can be utilized for outreach. Care must be culturally appropriate and affordable, and collaboration with health authorities and NGOs is vital for integration, resource allocation, and potential funding.

## Conclusion

In conclusion, our study highlights adolescents’ distinct preferences for health information sources and their interest in utilizing E-clinics in the West Bank, Palestine. Given the unique Palestinian context, adolescents’ healthcare needs and digital engagement may differ from those in Western communities. Consequently, the Ministry of Health and HCPs must take an active role in enhancing adolescent healthcare delivery. Policymakers should implement regulatory frameworks ensuring data security and patient confidentiality in digital healthcare. Investment in nationwide digital infrastructure, including improved Internet access in underserved areas, is crucial for equitable E-clinic adoption. Additionally, integrating E-clinic services into existing healthcare facilities and establishing feedback mechanisms to assess adolescent satisfaction and engagement can enhance long-term sustainability. Future research should include longitudinal studies to evaluate E-clinics’ long-term impact on adolescent healthcare and inform policy improvements.

## Supporting information

S1 File Dataset file. (ZIP)
