# Supplementary material for: A study on information perception and engagement, emphasizing the essential role of E-clinics among Palestinian adolescents
Source: PLoS One. 2025 Apr 29;20(4):e0322220. doi: 10.1371/journal.pone.0322220 (PMC12040143; doi:10.1371/journal.pone.0322220)
Supplement: S1 File — (ZIP) [file pone.0322220.s001.zip › metafile.docx]

**Title: Metafile description of the dataset in the supporting information file:**

Age: None

Age categories: cut off 16 years

1: < 16 years

2: > or = 16 years

Place of residence

1: Northern Governorates

2: Central Governorates

3: Southern Governorates

Gender:

1: Female

2: Male

Location:

1: City

2: Village

3: Refugee camp

Education_Level:

1: 8^th^ grade

2: 9^th^ grade

3: 10^th^ grade

4: 11^th^ grade

5: 12^th^ grade (Tawjihi – high school level)

Q2_1: Trust in healthcare services

1: Strongly disagree

2: Disagree

3: Not sure

4: Strongly agree

5: Agree

Trust in healthcare providers

1: Disagree

2: Not sure

3: Agree

Q2_2: I have complete trust in healthcare services

1: Strongly disagree

2: Disagree

3: Not sure

4: Strongly agree

5: Agree

Trust healthcare services:

1: Disagree

2: Not sure

3: Agree

Q3_1: health related information sources:

1: Internet

2: Family

3: friends

4: healthcare and social service providers

5: school health counselors

6: other sources

Sources_NEW: preferred sources of health information

1: Internet

2: Healthcare and social providers

3: Family and friends

4: Others

Q3_2: reasons to search for health information online

1: It is free

2: easy to use

3: Quick to use

4: The Internet provides privacy (no one knows me)

5: There is a lot of information online

6: To get different opinions

7: Other reasons

Reasons to use the Internet as a source of health information:

1: Convenient

2: Privacy

3: Availability of information

4: Others

Q4_1: my personal and medical information can be shared with others

1: Strongly disagree

2: Disagree

3: Not sure

4: Strongly agree

5: Agree

Q4_2: Are you concerned about the privacy and confidentiality of your health information

1: Very concerned

2: Somewhat concerned

3: Concerned

4: Not concerned

5: Not concerned at all

Privacy_Concerns

1: Disagree

2: Not sure

3: Agree

Q5_11: evaluation of the accuracy of online health information

1: not trustworthy at all

2: not very trustworthy

3: somewhat trustworthy

4: very trustworthy

Q5_12: social media

1: not trustworthy at all

2: not very trustworthy

3: somewhat trustworthy

4: very trustworthy

Q5_13: government websites (WHO, MOH …..)

1: not trustworthy at all

2: not very trustworthy

3: somewhat trustworthy

4: very trustworthy

Q5_14: Magazines and brochures

1: not trustworthy at all

2: not very trustworthy

3: somewhat trustworthy

4: very trustworthy

Q5_15: healthcare and social service providers

1: not trustworthy at all

2: not very trustworthy

3: somewhat trustworthy

4: very trustworthy

Q5_16: Radio and TV

1: not trustworthy at all

2: not very trustworthy

3: somewhat trustworthy

4: very trustworthy

Q5_2: ever used the Internet to search for health information

0: No

1: Yes

Q5_31: searching online takes a lot of effort to find the information I need

0: Disagree

1: Agree

Q5_32: I am concerned about the quality of information I find online

0: Disagree

1: Agree

Q5_33: The information I find online is difficult to understand

0: Disagree

1: Agree

Q6_1: Why do you think you can use the E-clinic

1: obtain information about a chronic illness

2: Get a calorie-related dietary plan

3: obtain information about sports and physical activities

4: receive reminders for medical prescriptions

5: receive reminders for medical check-ups

6: to maintain good health

Other purposes

Q6_2: why do you think you can use the E-clinic for

1: Verify the accuracy of medical information related to me

2: Manage my regular visits, medications, vaccinations, and other information

3: Know the results of your lab tests and other tests

4: Access instructions from your personal doctor

5: Receive reminders when you need medical check-ups

6: Renew monthly medication prescriptions

7: Schedule an appointment with a doctor

8: Send or receive emails from your doctor or nurse

9: Share your information with your family

10: I am not interested in using E-clinic

Q6_31: updating your health information using E-clinic

1: Strongly disagree

2: Disagree

3: Not sure

4: Strongly agree

5: Agree

Q6_32: I am concerned about the privacy of my personal and health information

1: Strongly disagree

2: Disagree

3: Not sure

4: Strongly agree

5: Agree

Q6_33: I am interested in using the E-clinic to manage my health and medical needs

1: Strongly disagree

2: Disagree

3: Not sure

4: Strongly agree

5: Agree

Q6_34: I like using the computers and the internet to search for health information

1: Strongly disagree

2: Disagree

3: Not sure

4: Strongly agree

5: Agree

Q6_35: Using e-clinics takes a lot of time

1: Strongly disagree

2: Disagree

3: Not sure

4: Strongly agree

5: Agree

Q6_4:E-clinics are benefit

0: Not beneficial

1: Beneficial

2: Very beneficial

Age_Scale:

1: less than 14

2: from 14.1 to 16

3: from 16.1 to 18

4: over 18 years

Descriptives:

Healthcare and social providers

Governorate websites

Magazines and brochures

Evaluation of the accuracy of health information

Radio and TV

Social media

E-clinics ensures privacy

1: Disagree

2: Not sure

3: Agree

E-clinic is beneficial

1: Disagree

2: Not sure

3: Agree

E-clinics take time and effort

1: Disagree

2: Not sure

3: Agree
